# Recent independent emergence of multiple multidrug-resistant *Serratia marcescens* clones within the United Kingdom and Ireland

**DOI:** 10.1101/gr.205245.116

**Published:** 2016-08

**Authors:** Danesh Moradigaravand, Christine J. Boinett, Veronique Martin, Sharon J. Peacock, Julian Parkhill

**Affiliations:** 1 Wellcome Trust Sanger Institute, Wellcome Genome Campus, Hinxton, Cambridgeshire CB10 1SA, United Kingdom;; 2 British Society for Antimicrobial Chemotherapy, Birmingham B1 3NJ, United Kingdom;; 3 Department of Medicine, University of Cambridge, Cambridge CB2 0QQ, United Kingdom;; 4 London School of Hygiene and Tropical Medicine, London, WC1E 7HT, United Kingdom

## Abstract

*Serratia marcescens*, a member of the Enterobacteriaceae family, is a Gram-negative bacterium responsible for a wide range of nosocomial infections. The emergence of multidrug-resistant strains is an increasing danger to public health. To design effective means to control the dissemination of *S. marcescens*, an in-depth analysis of the population structure and variation is required. Utilizing whole-genome sequencing, we characterized the population structure and variation, as well as the antimicrobial resistance determinants, of a systematic collection of antimicrobial-resistant *S. marcescens* associated with bloodstream infections in hospitals across the United Kingdom and Ireland between 2001 and 2011. Our results show that *S. marcescens* is a diverse species with a high level of genomic variation. However, the collection was largely composed of a limited number of clones that emerged from this diverse background within the past few decades. We identified potential recent transmissions of these clones, within and between hospitals, and showed that they have acquired antimicrobial resistance determinants for different beta-lactams, ciprofloxacin, and tetracyclines on multiple occasions. The expansion of these multidrug-resistant clones suggests that the treatment of *S. marcescens* infections will become increasingly difficult in the future.

The Gram-negative bacillus *Serratia marcescens* is a member of the Enterobacteriaceae that resides in environmental soil and water (Szewzyk et al. 1993). This species was initially considered nonpathogenic and due to its red pigment was used by the military to ascertain spread of bacteria in the natural environment after deliberate release (Mahlen 2011). However over recent decades, *S. marcescens* has been increasingly recognized as an important causative agent of opportunistic nosocomial infections (Mahlen 2011). *S. marcescens* has been isolated from a variety of sites including the respiratory tract (Lancaster 1962), bloodstream (Seeyave et al. 2006), and wounds. *S. marcescens* also has the potential to cause meningitis, particularly in patients with compromised immune systems (Mahlen 2011). Although existing reports suggest community acquisition and onset for some *S. marcescens* infections (Laupland et al. 2008; Mahlen 2011), the number of reports of *S. marcescens* infection in hospitals has seen a sharp rise. This has led to *S. marcescens* being largely associated with hospital-derived nosocomial infection. *S. marcescens* infections are more often associated with intensive care units, surgical wards, and dialysis units (Ringrose et al. 1968; Donowitz et al. 1979; Geiseler et al. 1982; Krishnan et al. 1991), and outbreaks of *S. marcescens* infection have been observed within a single hospital (Bullock et al. 1982; Su et al. 2003) and involving multiple hospitals within one city (Craven et al. 1977; Hejazi et al. 2000; Naumiuk et al. 2004).

*S. marcescens* associated with hospital outbreaks are frequently resistant to multiple antimicrobials. This species has intrinsic resistance to several antimicrobial groups, including some classes of beta-lactams and tetracyclines (Livermore et al. 2001; Stock et al. 2003). *S. marcescens* is intrinsically susceptible to other antimicrobial groups including quinolones and aminoglycosides, although chromosomal or plasmid-mediated resistance has been identified for some of these (Livermore et al. 2001; Stock et al. 2003). Like other nosocomial pathogens, the increasing pathogenicity of *S. marcescens* infections has been linked with the rise in the use of antimicrobials; however, the evidence supporting this hypothesis is not strong for *S. marcescens* (Mahlen 2011).

Among the major pathogenic Enterobacteriaceae, *S. marcescens* is one of the least well-studied to date, which has been mainly due to the recent recognition of the species as a pathogen. Whole-genome sequence data for *S. marcescens* from the environment and clinical samples have only recently become available (Chung et al. 2013; Liu et al. 2013; Iguchi et al. 2014; Wan et al. 2015). Taken together, these indicate that the *S. marcescens* genome is highly dynamic, which reflects the diversity of environmental niches that the bacterium occupies and the opportunistic pathogenic nature of the organism. In addition to bacteriocin and pigment genes, clinical isolates have been shown to harbor various beta-lactamases, including ampC beta-lactamase, metallo-beta-lactamase, and several putative multidrug efflux pumps (Chung et al. 2013; Liu et al. 2013; Iguchi et al. 2014; Wan et al. 2015). As with other Gram-negative nosocomial bacteria, antimicrobial resistance determinants are mainly located on the accessory elements of the *S. marcescens* genome (Chung et al. 2013; Liu et al. 2013; Iguchi et al. 2014; Wan et al. 2015).

Here, we utilized a national collection of multidrug-resistant *S. marcescens* isolated from bloodstream infections to perform a population-level study to elucidate the population structure of the bacterium. From this, we were able to estimate time of divergence of specific clones and recent nucleotide substitution rate and identify genetic mechanisms of antimicrobial resistance. Our results indicate that the *S. marcescens* population associated with antimicrobial-resistant bloodstream infections in the United Kingdom comprises a limited number of clades that have undergone recent near-simultaneous expansion. Recent inter- and intra-hospital transmissions were detected, and we demonstrated that antimicrobial resistance determinants have been independently acquired throughout the population.

## Results

**Figure 1.**
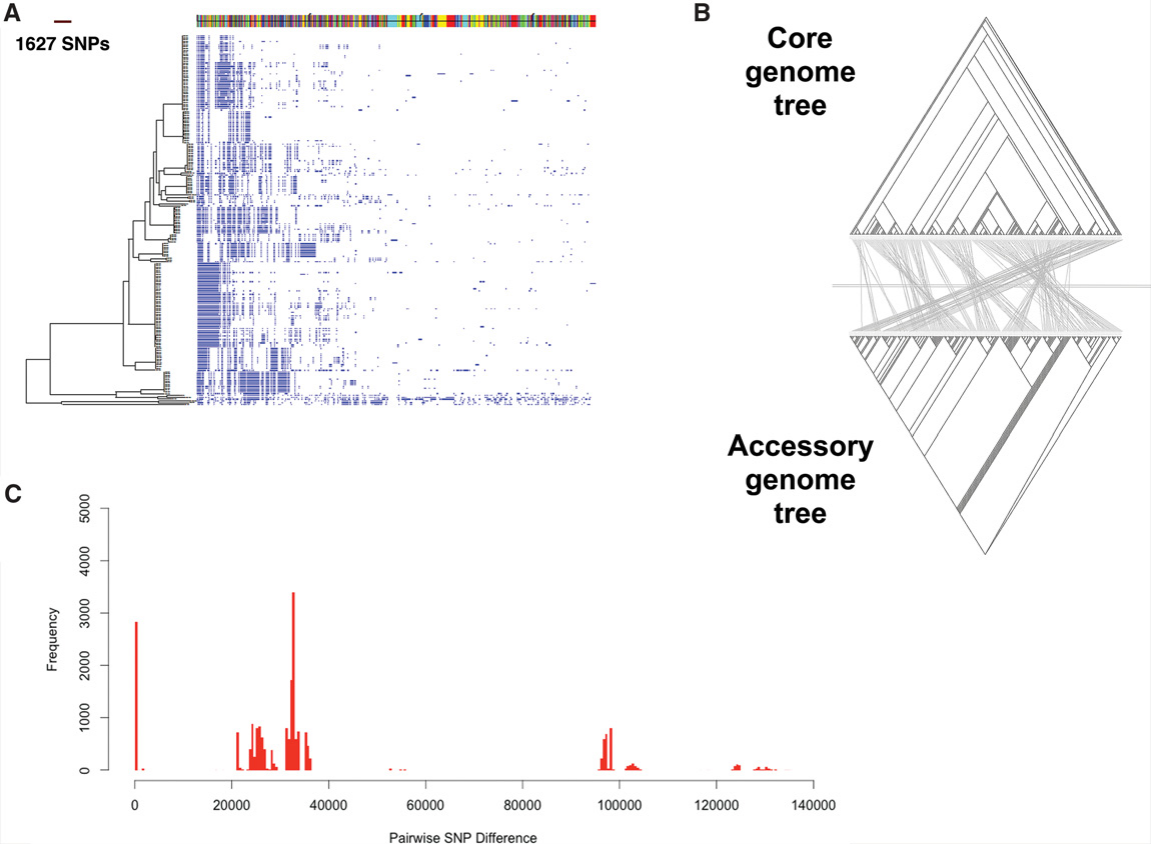
Pan-genome analysis of *Serratia marcescens* whole-genome sequences. (*A*) Maximum likelihood tree constructed from the core genome. On the *right*, the presence (blue) and absence (white) of accessory genome elements is shown. Accessory elements located on the same contig are shown as colored blocks along the *top* of the figure. (*B*) Comparison between the binary tree constructed from the absence and presence pattern of genes in the accessory genome (*bottom*) and the maximum likelihood tree constructed from variation in the core genome (*top*). The lines connect identical tips of the two trees. (*C*) A histogram of pairwise SNP distances between all 205 isolates. The distances are based on the core genome alignment.

We sequenced 205 multidrug-resistant (MDR) *S. marcescens* isolates associated with bloodstream infections in the United Kingdom and Ireland (UK&I) that had been systematically collected by the BSAC Resistance Surveillance Project between 2001 and 2011. Since *S. marcescens* belongs to the Enterobacteriaceae family, in which plasmids and mobile elements constitute an important part of the genome, we first identified the core genome (i.e., genes present in every isolate) and the accessory genome (i.e., genes variably present between isolates) (Fig. 1A). This identified a total of 13,614 genes (the pan-genome) consisting of 3372 core genes shared by >99% of the isolates, 342 soft core genes shared by 95%–99% of the population, 1526 shell genes shared by 15%–95% of the population, and 8347 cloud genes shared by <15% of the population. The numerous accessory genes underline the diversity of the *S. marcescens* species and the dynamic nature of its genome.

We then constructed a phylogenetic tree based on a core genome SNP alignment and compared this with the pattern of accessory genetic elements (Fig. 1A,B). The tree revealed a highly structured population consisting of distinct clades (Fig. 1A) with similar branch lengths. Moreover, each clade had a unique combination of accessory genes (Fig. 1A). Comparison between a tree constructed from the gene presence pattern in the accessory genome and the core genome tree suggested that each clade began with a specific set of accessory genes, with little change in their subsequent evolutionary history (Fig. 1B). The clonal structure of the population was also evident in the distribution of pairwise SNP distances of the core genomes (Fig. 1C). The distribution revealed that genetic distance across the phylogenetic tree was large, but that recent clonal expansion of a limited number of MDR clades has structured the distribution of diversity (Fig. 1C).

**Figure 2.**
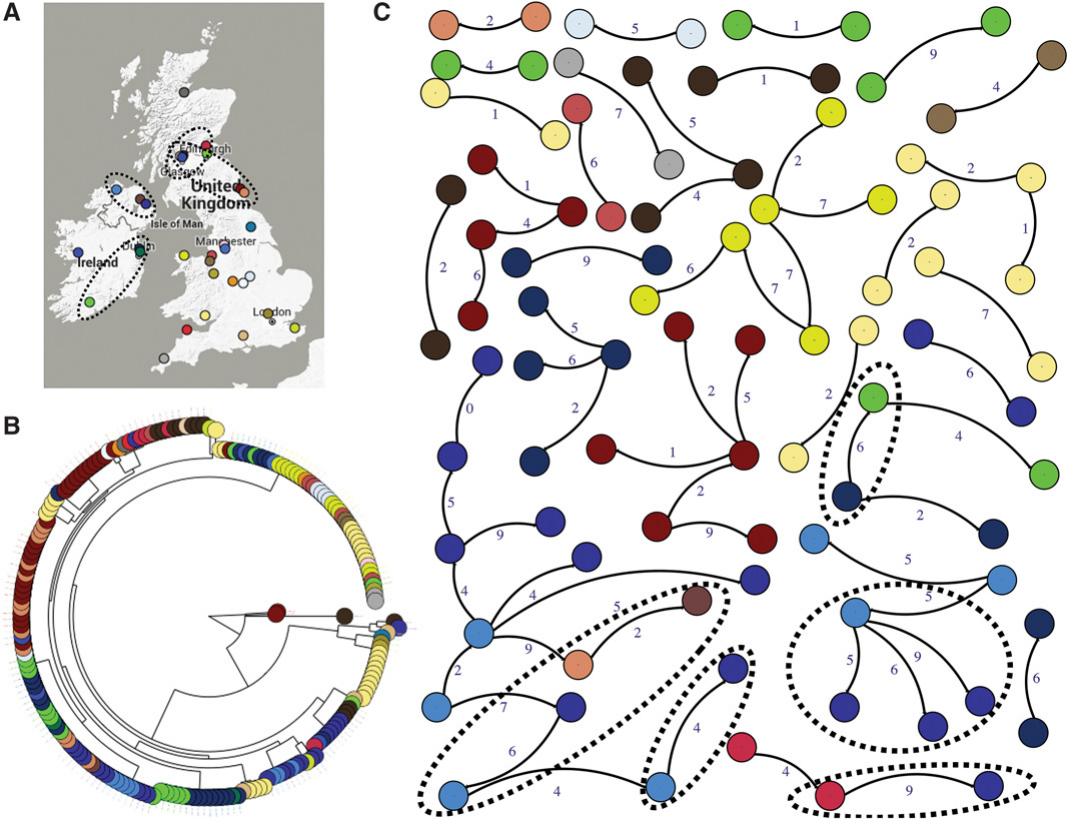
Geographical distribution and transmission network analysis. (*A*) Isolation sites across the UK&I; (*B*) maximum likelihood tree with isolates colored according to location. (*C*) The transmission network constructed for potential transmission events. Only edges of <10 SNPs are displayed, and singleton nodes are not shown. Colors correspond to hospitals. Dashed circles show potential between-hospital transmissions and the locations of those hospitals on the map. The numbers next to edges denote pairwise SNP distance between isolates.

A phylogenetic tree color coded by isolate origin showed some evidence for geographical clustering such that closely related isolates were found in some hospitals, although isolates from some hospitals were distantly related and resided in different parts of the tree (Fig. 2A). This suggests that *S. marcescens* MDR clades are partly hospital (or region)-specific, but that transmission between regions or hospitals has occurred. Comparing the distribution of pairwise SNP distance with geographical distance did not show any relationship between geographical and SNP distance for large SNP distances (i.e., >10) (Supplemental Fig. S1). However, for closely related isolates, there was a relationship between geographical distance and SNP distance (Supplemental Fig. S1), supporting the hypothesis that *S. marcescens* MDR clades have recently spread between hospitals. To examine this, we constructed a transmission network for isolates selected as being putatively associated with transmission events in the past 5–10 yr (Methods) (Fig. 2B). In addition to recent transmission within hospitals, we also identified some cases of inter-hospital transmission, mainly between geographically linked hospitals.

**Figure 3.**
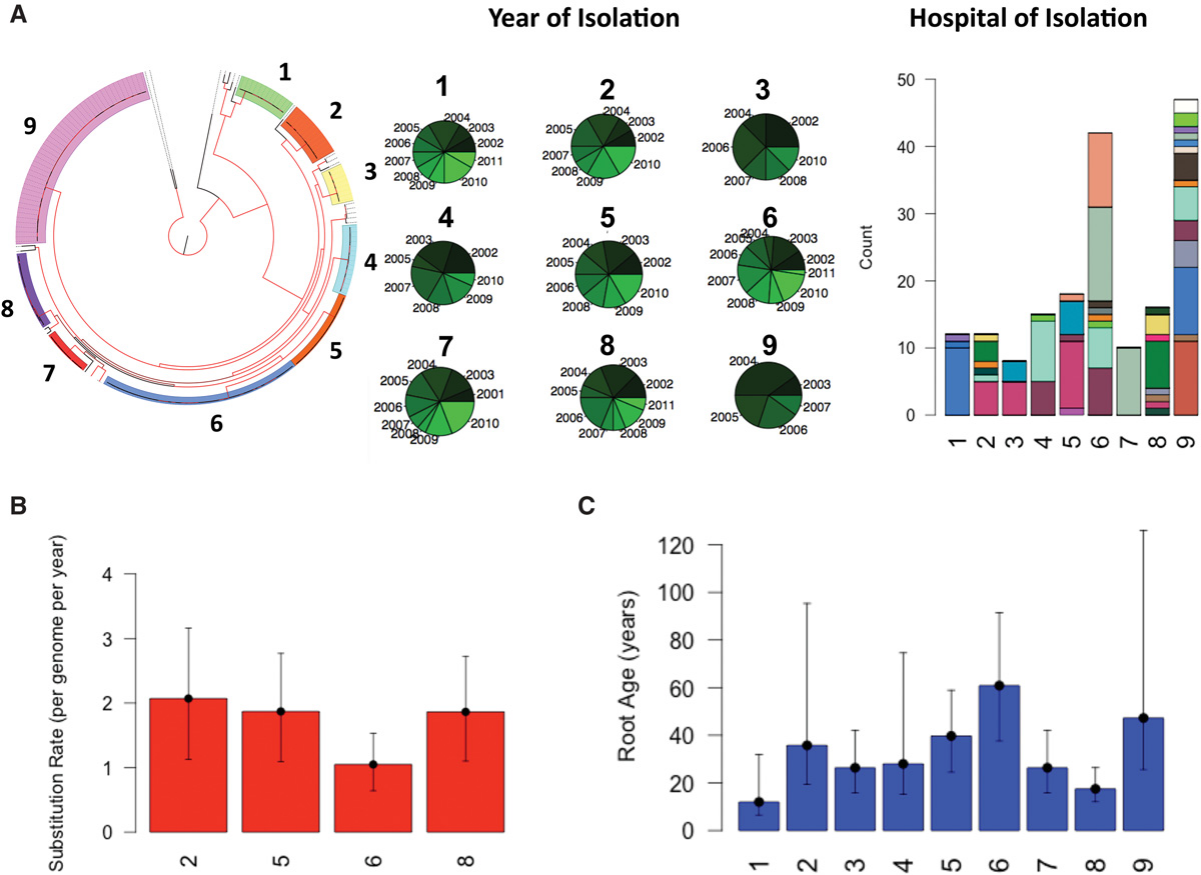
Temporal, geographical and phylogenetic clustering of isolates on the tree. (*A*) Phylogenetic tree with isolates colored according to cluster. The nodes with bootstrap support >90 are shown in red. The pie charts show the distribution of years of isolation and the color intensity corresponds to the temporal order, namely, the older the year of isolation the darker the color. The stacked bar plot shows the distribution of hospitals across the major clades on the phylogenetic tree. Each color corresponds to one hospital. (*B*) The estimated substitution rate for the clusters with significant temporal signal. Error bars show the 95% confidence intervals. The mutation rate is obtained by taking the average of three independent BEAST runs. (*C*) The estimated age of the MRCA for each cluster. Error bars correspond to 95% confidence interval for clusters shown in *B*. For the clusters without significant temporal signals, i.e., clusters 1, 3, 4, 7, and 9, we calculated the mean root age by using the mean substitution rate for clusters 2, 5, 6, and 8. For error bars, we considered the substitution rates obtained for maximum and minimum of 95% confidence intervals for clusters 2, 5, 6, and 8.

We then used a distance-based clustering algorithm to delineate the major clades within the population and found that isolates fell into nine major clades (Fig. 3A). All clades contained isolates collected over a period of 10 yr, but these exhibit varying degrees of geographical distribution. Specifically, although the majority of isolates in major clades 1, 3, 4, 5, and 7 were from a single hospital, the other major clades contain isolates from multiple hospitals (Fig. 3A). This supports the hypothesis of independent origins of largely region-specific *S. marcescens* major clades, with some geographical transmission. Utilizing the variation in time of isolation for isolates in each major clade, we attempted to calculate the recent nucleotide substitution rate and the age of each major clade. The Bayesian analysis revealed that for major clades 2, 5, 6, and 8 in which the temporal signal was significant (Methods), three major clades had a substitution rate of ∼5.8 × 10^−7^ SNPs/site/year (about two SNPs per genome per year), and each major clade had a most recent common ancestor (MRCA) between 20 and 40 yr ago (Fig. 3B,C). Clade 6 has a similar, but slightly slower substitution rate of 2.9 × 10^−7^ SNPs/site/year (approximately one per genome per year) and had an MRCA ∼80 yr ago (Fig. 3B,C). Applying these substitution rates to the other major clades allowed us to estimate MRCAs of between 10 and 80 yr ago. The recent and near-simultaneous emergence of these major MDR clades suggests that nosocomial MDR *S. marcescens* infection in the UK&I have been largely driven by a limited number of dominant transmissible MDR clones that might have been selected for by antimicrobial use. To determine the contribution of recombination to the intra-major clade diversity, we counted the number of recent recombination incidents for each major clade. We found that recombination was absent in major clades 8 and 9 and had occurred at an average rate between 0.01 and 0.07 events per genome per year for the other major clades. Since these values are much smaller than the substitution rates, we conclude that recombination has played only a minor role in shaping the diversity within major clades.

In a previous genomic study of *S. marcescens* clinical strain SM39 and insect strain Db11, plasmids pSMC1 and pSMC2 carrying beta-lactam and multiple other drug resistance genes were identified (Iguchi et al. 2014), but none of the isolates in our collection carried either of these plasmids. However, a search of the PlasmidFinder database for plasmids revealed that some isolates had replicons of some plasmids that were very similar to those from other Gram-negative species including *Escherichia coli* and *Klebsiella pneumoniae,* which were present in isolates scattered throughout the tree (Supplemental Fig. S2). The plasmids identified in our collection included pJEG011 and pIGMS32 plasmids, which have been reported previously in collections associated with *K. pneumoniae* outbreaks and are known to be capable of transferring between distantly related bacteria (Espedido et al. 2013). Moreover, the plasmids pNDM-KN, R46, and pFSEC-01, which we found in our collection, have been shown to carry several beta-lactamase genes (Supplemental Fig. S2; Brown and Willetts 1981; Zhang et al. 2016).

We then considered antimicrobial resistance for isolates in our collection (Supplemental Figs. S3, S4). A pairwise correlation analysis between the MICs for different antimicrobials showed a predictably strong correlation for three tetracyclines (tetracycline, minocycline, and tigecycline) and between some beta-lactams, including cefotaxime and piperacillin-tazobactam (Supplemental Fig. S4A). It is expected that resistance determinants can confer resistance to multiple antimicrobials with similar mechanisms of action but there are some interesting additional correlations and anti-correlations in the matrix that may be worthy of further study (Supplemental Figs. S4, S5). Based on resistance to antimicrobials with defined clinical breakpoints (Supplemental Figs. S4B, S5B), the collection was extensively resistant to several beta-lactams, including amoxicillin (S:0, I:0, R:205), cefuroxime (S:3, I:0, R:202), amoxicillin-clavulanate (S:0, I:0, R:205), cefotaxime (S:2, I:1, R:182), piperacillin-tazobactam (S:6, I:32, R:167), and ceftazidime (S:153, I:39, R:13). Intermediate resistance was more common to the fluoroquinolone ciprofloxacin (S:15, I:64, R:126) and tigecycline (S:132, I:57, R:13). The majority of isolates were susceptible to imipenem (S:203, I:2, R:0) and gentamicin (S:188, I:2, R:15).

We then undertook a genetic analysis of resistance mechanisms for specific drugs. Resistance to fluoroquinolones commonly arises as a result of alterations in the target enzymes DNA gyrase and topoisomerase IV (Rodríguez-Martínez et al. 2011). In particular, mutations at certain sites in *gyrA* largely account for ciprofloxacin resistance in Enterobacteriaceae (Dimri and Das 1990; Deguchi et al. 1997; Weigel et al. 1998). We noted that mutations at Ser-83 occurred in 89 isolates across the phylogenetic tree and were strongly correlated with MIC values (Supplemental Fig. S6; Supplemental Table S2). This mutation is the first step in selection for elevated resistance to fluoroquinolones in *E. coli* (Tankovic et al. 1994). Furthermore one isolate with an extremely high MIC appeared to harbor the known Asp-87 resistance mutation in *gyrA* (Supplemental Fig. S6; Weigel et al. 1998). In addition to these chromosomal mutations, the acquisition of a variant in the theta subunit of DNA polymerase III, seemingly inserted within the chromosome, by some subclades is strongly associated with elevated MIC values for ciprofloxacin (Supplemental Fig. S6). Although its function is not fully elucidated, this subunit is thought to be implicated in the stabilization of the epsilon subunit of DNA Polymerase III, which is involved in DNA proofreading, especially under extreme conditions (Taft-Benz and Schaaper 2004). Further analysis is required to elucidate the connection between ciprofloxacin and the theta subunit.

Resistance to aminoglycosides is mediated through the action of aminoglycoside modifying enzymes, i.e., acyltransferases, adenylyltransferase, or phosphotransferases. All isolates harbored acyltransferase *aac(6′)-Ic*, a well-known chromosomally encoded resistance gene (Champion et al. 1988), and some isolates carry *aadA*, although these genes did not appear to affect susceptibility of the population to gentamicin. However, reduced susceptibility to gentamicin occurred independently in some subclades of the major clades across the tree and could be attributed to the presence of one of aminoglycoside phosphotransferase *aph*(3′), acetyltransferase *aac*, or adenylyltransferase *aadb* (Supplemental Fig. S6). These genes seem to be located on wide host range plasmids originally identified in *K. pneumoniae*: The *aadB* gene is located on pMU407 shown in Supplemental Figure S2. The *aac(3)-I* gene occurs in the context of an IncFII group conjugative plasmid, previously reported as a multidrug resistance gene carrier (Rodríguez et al. 2010), and the *aph*(3′) and *aac(3)-II* genes are situated on various *K. pneumoniae* multidrug resistance plasmids.

*S. marcescens* collections have been reported to be uniformly resistant to tetracycline (Livermore et al. 2001; Stock et al. 2003). Despite this, we found a significant variation in MIC values for tetracyclines for our isolates (Supplemental Fig. S5A,B). Increased resistance to tetracycline occurred independently in four subclades across the phylogenetic tree and was linked with the presence of efflux pumps *tetA5* and *tetA3*, both of which have been reported as resistance determinants (Supplemental Fig. S6; Bollmann et al. 1989). The *tetA5* gene also accounted for the elevated resistance to minocycline in 16 samples and appears to be inserted within the chromosome (Table 1). The *tetA3* efflux gene, however, was found in the context of the pIGT15 plasmid, which originates from a clinical *Escherichia coli* isolate (Adamczuk et al. 2015). The presence of tetracycline transporter *tet41* in some isolates across the tree did not affect the susceptibility to tetracylines.

**Table 1.**
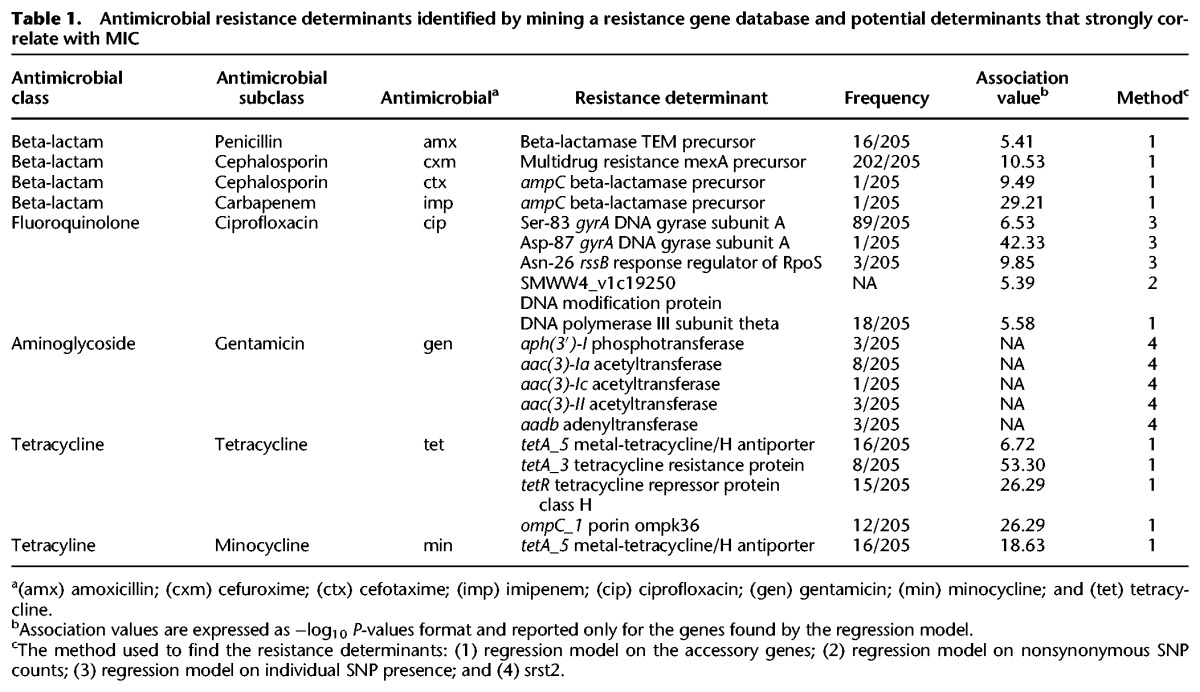
Antimicrobial resistance determinants identified by mining a resistance gene database and potential determinants that strongly correlate with MIC

Among the beta-lactam antimicrobials that we studied here, resistance in *S. marcescens* has been reported previously to ampicillin, amoxicillin, amoxicillin-clavulanate, and cefuroxime (Livermore et al. 2001; Stock et al. 2003). This is largely attributed to the plasmid-mediated extended-spectrum beta-lactamases (ESBLs) and the chromosomal *ampC* beta-lactamase, which can be induced by cefoxitin, imipenem, and amoxicillin as well as by some cephalosporins including ceftazidime, cefotaxime, and cefuroxime (Hanson and Sanders 1999; Naumiuk et al. 2004). Although every isolate in our collection contained *ampC*, only two samples were phenotypically detected as ESBL positive. Although one of the ESBL-positive isolates appears to carry a copy of *bla-SHV*, located in the context of a broad host range *Klebsiella pneumoniae* plasmid (pKEC-dc3), we did not identify any ESBL gene in the other isolate. The rarity of ESBLs indicates that resistance to cephalosporins and penicillin is predominantly mediated by chromosomal genes. Despite this, variation in the MIC values for some cephalosporins suggests that part of the population has acquired additional resistance mechanisms. In Table 1, we report some potential elements strongly associated with elevated MICs. Among beta-lactam antimicrobials, the population is extensively susceptible only to imipenem (Supplemental Figs. S4, S5B), which can be explained by the lack of well-known carbapenamases such as the KPC enzyme (Cai et al. 2008) and the metallo-beta-lactamase *imp* enzyme (Osano et al. 1994). The isolate with a high MIC for imipenem appeared to harbor an extra copy of *ampC*, suggesting the overproduction of AmpC was responsible for imipenem resistance in this isolate, as previously reported (Jacoby 2009; Lister et al. 2009). Table 1 presents some of the putative antimicrobial resistance determinants that exhibit a strong association with MICs, and a full list of gene/SNP hits, strongly associated with resistance, is provided as Supplemental Tables S2–S4.

## Discussion

We have described an in-depth analysis of a collection of MDR *S. marcescens* from bloodstream infections in the UK&I, in which we elucidated the population structure and dynamics, as well as antimicrobial resistance determinants. *S. marcescens* was previously believed to be a nonpathogenic environmental species but has been recognized as an increasingly important opportunistic pathogen over the past few decades (Mahlen 2011). The large accessory genome and genomic flexibility is likely to be important for its ability to act as an opportunistic pathogen (Aujoulat et al. 2012).

Our results revealed that the MDR *S. marcescens* population responsible for bloodstream infections in UK&I hospitals has a strongly clonal structure, with a limited number of clones having emerged within the last few decades. Utilizing the fine resolution of whole-genome sequencing, we were able to identify potential transmissions of these MDR clones between and within hospitals. In spite of several cases of within-hospital transmissions, we found that pathogens within some hospitals had very different origins, implying either that some patients acquired the infection outside hospital or that inter-hospital transmission had taken place. More detailed tracking of all *S. marcescens* infections within a hospital or healthcare system would shed more light on the structure of the underlying transmission network.

The rapid increase of nosocomial *S. marcescens* infections and the rise in the use of antimicrobials indicates that recent antimicrobial therapy might have initiated the emergence or accelerated the spread of MDR *S. marcescens* clones. This hypothesis appears to hold to some extent for our isolates as the increased MIC values occur uniformly across the phylogenetic tree, and identical antimicrobial resistant determinants at both SNP and gene levels have been acquired multiple times across the tree. This is further supported by the parallel emergence of resistance variants through the acquisition of antimicrobial resistance genes or mutations, as shown in Supplemental Figure S5. Furthermore the pattern of acquisition of resistance determinants across the phylogenetic tree appeared to be different for various genes. In some cases (e.g., tetA-5), all isolates within a major clade acquired the resistance genes, although in many cases (e.g., *accA* and *aadB* genes), several subclades of major clades have gained the gene. The latter mode of resistance acquisition by subclades of major clades suggests that either the subclades still undergo changes in response to antimicrobial treatment or other evolutionary forces contribute to variation in resistance levels.

The current therapy for *Serratia* infections includes an aminoglycoside plus a beta-lactam and/or a carbapenem, since the single use of a beta-lactam can select for resistant strains. Our findings of correlated resistance demonstrate that the concurrent application of most beta-lactams may be ineffective and only gentamicin, imipenem, and to a lesser extent, tigecycline, appear to still offer effective treatment (Hope et al. 2006; Harris and Ferguson 2012). Gentamicin was commonly used during the 1970s, and as a result, resistance quickly arose at that time (Yu et al. 1979). However, the increasing use of other antimicrobials, such as ciprofloxacin and cephalosporins, led to a reduction in the use of gentamicin. The uniform lack of resistance in the clones identified here suggests that the earlier gentamicin-resistant *S. marcescens* may have belonged to a clone (or clones) that has subsequently been lost from the population by competition with extant clones, although it is possible that the current clones may once have been gentamicin resistant. The reuse of gentamicin in clinical practice can however accelerate the spread of the few antimicrobial resistance determinants we identified, and this could result in outbreaks of aminoglycoside resistant *S. marcescens* as has been observed recently (Mlynarczyk et al. 2007; Ivanova et al. 2008).

Compared to other major nosocomial pathogens, less attention has been paid to *S. marcescens* infections due to its lower prevalence. Moreover, no genetic typing method is available for *S. marcescens*. Using whole-genome sequencing has allowed us to demonstrate that most MDR *S. marcescens* in UK&I hospitals belonged to a limited number of clones of recent origin, reminiscent of the much better studied clonal structure of methicillin resistant *Staphylococcus aureu*s (MRSA) (Reuter et al. 2016). One question that is not answered by this study is whether antimicrobial-susceptible *S. marcescens* infections are also caused by recent clones, or whether any representative of the broader species is capable of opportunistic infection. A global understanding of the evolution of antimicrobial resistance and dissemination of nosocomial *S. marcescens* could be attained using a broader collection from various sources, including a wide geographical distribution, different infection sources (e.g., wounds or urinary tract infections), and community/nosocomial origin, as has been done for other Enterobacteriaceae (Holt et al. 2015).

## Methods

### Isolates and antimicrobial susceptibility testing

The study was approved by the National Research Ethics Service (ref: 12/EE/0439) and the CUH Research and Development (R&D) Department. A national collection of 205 *S. marcescens* isolates was provided by the British Society for Antimicrobial Chemotherapy (BSAC) Resistance Surveillance project (http://www.bsacsurv.org) and consisted of isolates submitted to a bacteraemia surveillance program between 2001 and 2011 by 31 hospitals across the United Kingdom and Ireland. Multidrug resistance (MDR) was defined as acquired nonsusceptibility to at least one agent in three or more antimicrobial categories (Magiorakos et al. 2012), and isolates in this collection were selected based on the presence of phenotypic resistance to at least one antimicrobial in three of the following groups: penicillins, carbapenems, cephalospsorins, tetracyclines, aminoglycoside, and fluoroquinolones. A list of isolates is provided in Supplemental Table S1. The minimum inhibitory concentration (MIC) for each antimicrobial agent was determined using the BSAC agar dilution method. Antimicrobials included beta-lactams (penicillin: amoxicillin; cephalosporins: cefuroxime, cefoxitin, cefotaxime, ceftazidime, and piperacillin-tazobactam and amoxicillin-clavulanic acid), tetracyclines (minocycline, tetracycline, and tigecycline), aminoglycosides (gentamicin), and fluoroquinolones (ciprofloxacin). We compared the distribution of MIC values of our samples with those from the European Committee on Antimicrobial Susceptibility Testing (EUCAST). These, along with the clinical susceptibility breakpoints used in our study were downloaded from the EUCAST website (http://www.eucast.org) on May 1, 2016.

### Sequencing, pan-genome analysis and phylogeny

DNA was extracted using QIAxtractor (Qiagen), according to the manufacturer's instructions. Library preparation was conducted according to the Illumina protocol, and sequencing was performed on an Illumina HiSeq2000 with a read length of 2 × 100 bp. Ninety-six samples were multiplexed per lane to give an average depth of coverage of 79-fold. The raw sequences and assembled data have been deposited in the ENA under the accession numbers shown in Supplemental Table S1. Paired-end sequence reads were assembled using an in-house assembly and improvement pipeline based on Velvet (Zerbino and Birney 2008) and annotated with Prokka, which takes fragmented de novo assemblies (Seemann 2014). To identify core and accessory genes, we used the annotated assemblies produced by Prokka as input to Roary, a tool that rapidly builds large-scale pan-genomes (Page et al. 2015). Isolates were then clustered based on the presence of various genes in the accessory genome, with the contribution of isolates to the graph weighted by cluster size. The maximum likelihood phylogenetic tree from the core genome was built with RAxML using a general time reversible (GTR) evolutionary model and a gamma correction for among site rate variation (Stamatakis 2014). We conducted one hundred bootstrap replicates to quantify the significance of nodes in the maximum likelihood tree. We used in-house tools, Microreact (http://microreact.org) and *Dendroscope* (Huson and Scornavacca 2012) to visualize the results.

### Recombination analysis

We used Gubbins to identify regions of the genomes that had undergone recent homologous recombination. The tool detects recombination based on the elevated SNP density, which identifies homologous recombination from within or outside the tree (Croucher et al. 2015). For nucleotide substitution rate estimation, we constructed a maximum likelihood phylogeny from the multiple alignment file that Gubbins produces after excluding the recombinant regions.

### Transmission analysis and cluster analysis

We constructed the network of potential recent transmission events using the R package adegene, which maximizes the likelihood of the network constructed from pairwise SNP distances between samples (Jombart and Ahmed 2011; Jombart et al. 2011). We excluded edges corresponding to more than 10 SNPs, which given the estimate of the recent substitution rate of the core genome of *S. marcescens*, corresponds to SNPs accumulated over ∼5 to 10 yr (Results). We identified clusters of samples within our collection using an unsupervised clustering algorithm in the adegene package. The tool takes the pairwise SNP distance matrix and a SNP cut-off and generates the clusters. This means that for larger cut-off values, fewer clusters are produced. To find the best cut-off for clustering, we screened the SNP cut-off values from 0, which gives 205 clusters, to very large values, which give one cluster. After excluding five outliers that are too far from the rest of the samples, we identified 26 groups, which remained robust against changing cut-off values for 102,322 SNPs. We considered only the nine clusters with more than five members for substitution rate analysis. These nine clusters include 178 isolates in total.

### Phylogenetic analysis and mutation rate calculation

To estimate recent nucleotide substitution rate, we constructed multiple alignments for each cluster after eliminating recombined regions and then reconstructed the maximum likelihood phylogenetic tree. We used this to plot root-to-tip distance versus year of isolation for each sample. To evaluate the significance of the temporal signal in each cluster, we performed 10,000 bootstraps with randomized years to obtain a distribution for *R*-squared values and compared the real *R*-squared values with the distribution. We then estimated the substitution rate and the age of the root node with BEAST v1.7 (Drummond and Rambaut 2007) for the clusters in which temporal signals within the data were found to be significant (90% confidence interval).

We examined various models including a strict molecular clock, a GTR model with a gamma correction for among site variation, and relaxed exponential model with constant population model (the models yielded similar results). For each parameter set, three independent chains were conducted for 100 million generations with sampling every 10 generations. Subsequently, convergence was assessed for each model by checking that ESS values were greater than 200 for key parameters. We also checked that independent runs had converged on similar values. A burn-in of 10 million states was left out from each of the three independent runs of this model. We then combined the results from those runs with the logcombiner program from the BEAST package. We took the age of the root as the height of the root of the Maximum Clade Credibility (MCC) tree reconstructed by combining trees using the tree annotator program from the BEAST package.

### Regression analysis and identification of antimicrobial resistance determinants

To identify antibiotic resistance genes in the isolates, we used srst2 (Inouye et al. 2014) to map short reads to known resistance genes in the Resfinder data set of the srst2 package (coverage cut-off 90%). As a complement to this approach, we utilized the MIC values to identify putative antibiotic resistance determinants that are associated with elevated MIC values. To this end, we used multiple regression approaches, i.e., identifying genes in which the number of nonsynonymous SNPs strongly correlates with resistance level; identifying genes in the accessory genome, the presence of which strongly correlates with resistance level; and finally identifying SNPs that strongly correlate with resistance levels. By doing so, we aimed to find not only already well-known resistance genes but also any putative resistance determinant that might be implicated in increasing resistance to a particular antimicrobial.

We directly used MIC values, instead of categorical resistant/susceptible status, as the predictor parameter in the regression analyses for two reasons: First, MIC values allowed us to address a more general question of what elements increase resistance levels. Second, the higher variation within MIC values than in the categorical resistance status provides the regression model with more information to yield more significant results (the variance of MIC values for some antimicrobials was not sufficiently large for regression analysis).

We performed regression analysis to single out potential resistance genes by taking the presence and absence of genes in the accessory genome as the predictor and MIC values as the dependent parameter. We then obtained the list of hits, i.e., genes with association value (*P*-value) <10^−5^, and excluded genes, such as phage genes, that were highly unlikely to play a role in resistance from the list of hits. Subsequently, we screened the list to find putative resistance genes. Although this approach helped us to find some potential resistance genes for some antimicrobials, we did not identify any gene for a few antimicrobials. This might be due to nongenetic or complex genetic effects, not studied here, on variation in MIC values, or the signal not being sufficiently strong for a significant result to be obtained with the cut-off used.

To conduct the SNP based association studies, we first mapped the short reads against the reference genome *S. marcescens* WW4 (accession number: CP003959) using SMALT v0.7.4 (https://www.sanger.ac.uk/resources/software/smalt/). SNPs were then annotated using a combination of SAMtools mplieup (Li et al. 2009) and BCFtools as detailed in Harris et al. (2010). The SNP association regression model identified SNPs that were strongly correlated with MIC values. In this analysis, sites with >10% unknown SNPs were excluded. In our second SNP-based analysis, we attempted to find genes with a strong association between their variation pattern and MIC. To this end, we computed the correlation between the number of nonsynonymous mutations and MIC for each gene in our database. As with the accessory gene-based model, we set a stringent *P*-value cut-off of <10^−5^ to obtain a list of hits. We used the SNP association method primarily for ciprofloxacin, resistance against which is primarily caused by mutations in chromosomal genes. In all the aforementioned regression models, we corrected and accounted for population structure by incorporating the clusters previously identified on the phylogenetic tree into the model.

## Data access

The sequence data for the isolates from this study have been submitted to the European Nucleotide Archive (ENA; http://www.ebi.ac.uk/ena) under accession numbers PRJEB5065 and ERP004424. The sequences of the genes in the pan-genome have been submitted to the Mendeley database (https://data.mendeley.com/) (Moradigaravand 2016).
